# Crystal structure of *N*,*N*′-(1,2-phenyl­ene)bis­(2-chloro­acetamide)

**DOI:** 10.1107/S2056989015000304

**Published:** 2015-01-14

**Authors:** Javaria Tariq, Shahzad Murtaza, Muhammad Nawaz Tahir, Muhammad Zaheer

**Affiliations:** a Department of Chemistry, Institute of Chemical and Biological Sciences, University of Gujrat, Gujrat 50700, Pakistan; b Department of Physics, University of Sargodha, Sargodha, Punjab, Pakistan

**Keywords:** crystal structure, 2-chloro­acetamide, secondary amide groups, hydrogen bonding

## Abstract

In the title compound, C_10_H_10_Cl_2_N_2_O_2_, the secondary amide groups are differently twisted relative to the benzene ring, with dihedral angles between the respective planes of 21.03 (2) and 81.22 (2)°. In the crystal, the mol­ecules are connected by N—H⋯O and C—H⋯O hydrogen bonds, forming a two-dimensional polymeric network parallel to (001). One of the amide carbonyl O atoms accepts two H atoms in N—H⋯O and C—H⋯O inter­actions, forming an *R*_2_^2^(6) ring motif.

## Related literature   

For the structure of *N*,*N*′-phenyl­enebisacetamide, see: Shivanyuk *et al.* (2000 ▸).
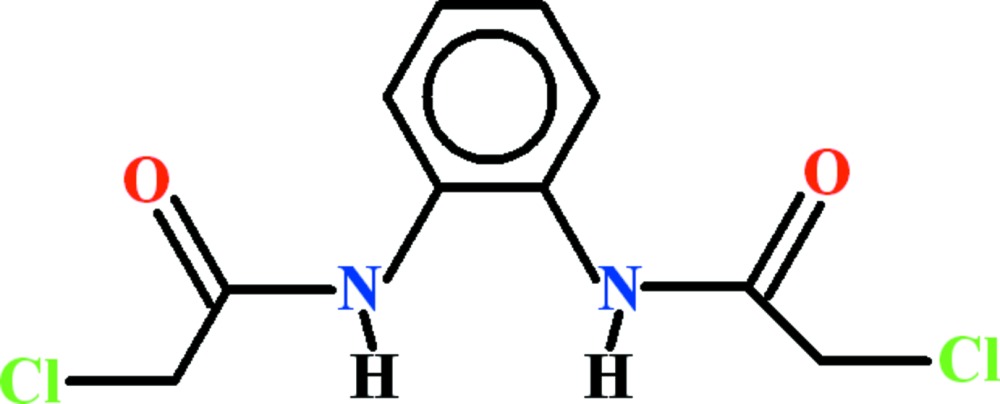


## Experimental   

### Crystal data  

C_10_H_10_Cl_2_N_2_O_2_
*M* *_r_* = 261.10
Monoclinic, 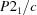

*a* = 4.5731 (4) Å
*b* = 14.3260 (16) Å
*c* = 16.7472 (15) Å
β = 95.611 (5)°
*V* = 1091.92 (18) Å^3^
*Z* = 4
Mo *K*α radiation
μ = 0.58 mm^−1^
*T* = 296 K
0.40 × 0.22 × 0.16 mm

### Data collection  

Bruker Kappa APEXII CCD diffractometer
Absorption correction: multi-scan (*SADABS*; Bruker, 2005 ▸) *T*_min_ = 0.803, *T*_max_ = 0.911
8308 measured reflections
2154 independent reflections
1685 reflections with *I* > 2σ(*I*)
*R*_int_ = 0.031

### Refinement  

*R*[*F* ^2^ > 2σ(*F* ^2^)] = 0.035
*wR*(*F* ^2^) = 0.083
*S* = 1.03
2154 reflections
145 parameters
H-atom parameters constrained
Δρ_max_ = 0.25 e Å^−3^
Δρ_min_ = −0.28 e Å^−3^

### 

Data collection: *APEX2* (Bruker, 2007 ▸); cell refinement: *SAINT* (Bruker, 2007 ▸); data reduction: *SAINT*; program(s) used to solve structure: *SHELXS97* (Sheldrick, 2008 ▸); program(s) used to refine structure: *SHELXL97* (Sheldrick, 2008 ▸); molecular graphics: *ORTEP-3 for Windows* (Farrugia, 2012 ▸) and *PLATON* (Spek, 2009 ▸); software used to prepare material for publication: *WinGX* (Farrugia, 2012 ▸) and *PLATON*.

## Supplementary Material

Crystal structure: contains datablock(s) I, global. DOI: 10.1107/S2056989015000304/gk2623sup1.cif

Structure factors: contains datablock(s) I. DOI: 10.1107/S2056989015000304/gk2623Isup2.hkl

Supporting information file. DOI: 10.1107/S2056989015000304/gk2623Isup3.cml

**Fig. 1.**
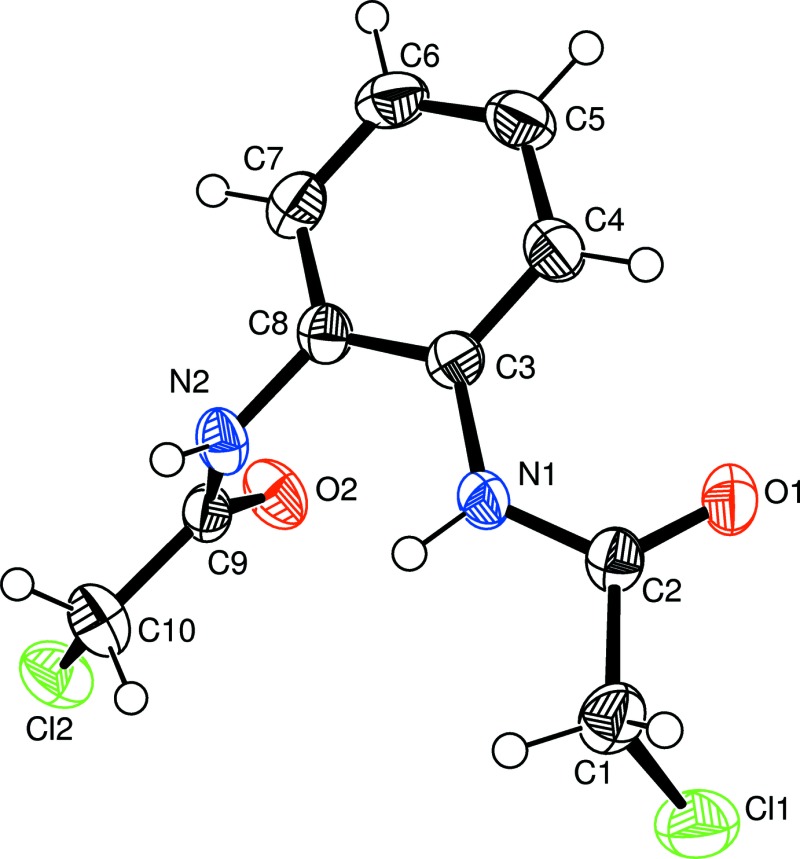
Molecular structure with displacement ellipsoids drawn at the 50% probability level. H-atoms are shown as small spheres of arbitrary radii.

. DOI: 10.1107/S2056989015000304/gk2623fig1.tif
Mol­ecular structure with displacement ellipsoids drawn at the 50% probability level. H-atoms are shown as small spheres of arbitrary radii.

**Fig. 2.**
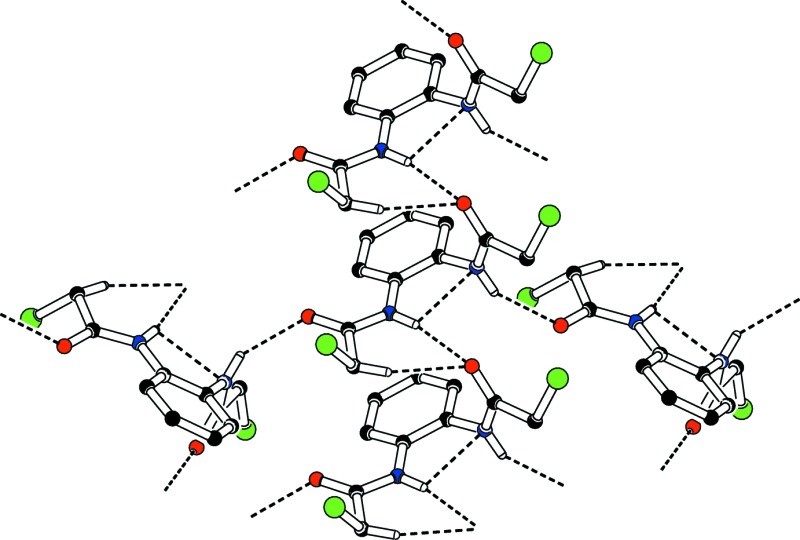
Two dimensional polymeric network fomed via hydrogen bonds. The H-atoms not involved in hydrogen bonding are omitted for clarity.

. DOI: 10.1107/S2056989015000304/gk2623fig2.tif
Two dimensional polymeric network fomed via hydrogen bonds. The H-atoms not involved in hydrogen bonding are omitted for clarity.

CCDC reference: 1042462

Additional supporting information:  crystallographic information; 3D view; checkCIF report

## Figures and Tables

**Table 1:** Hydrogen-bond geometry (, )

*D*H*A*	*D*H	H*A*	*D* *A*	*D*H*A*
N1H1O2^i^	0.86	2.16	3.003(2)	168
N2H2O1^ii^	0.86	2.23	3.004(2)	150
C1H1*A*O2^i^	0.97	2.44	3.333(3)	153

Symmetry codes: (i) 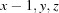; (ii) 
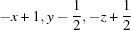
.

## supplementary crystallographic information

### S1. Comment

The title compound has been synthesized to check its antimicrobial activity owing to the concept that amide moiety is an important part of different drugs. The title molecule is shown in Fig. 1.

The benzene-1,2-diamine (C3—C8/N1/N2; A) unit is planar with r. m. s. deviation of 0.0084 Å. The attached chloroacetyl groups differ structurally. The groups B (C1/C2/O1) and C (C9/C10/O2) form with the fragment A the dihedral angles of 21.0 (2)° and 82.78 (13)°, respectively. The molecules are connected by N-H···O and C-H···O hydrogen bonds to form a two dimensional polymeric network parallel to (0 0 1) (Table 1, Fig. 2). In closely related N,N'-phenylenebisacetamide (Shivanyuk *et al.*, 2000) a one dimensional ribbon is formed.

### S2. Experimental

Benzene-1,2-diamine (0.1 g, 0.925 mmol) was dissolved in chloroform (10 ml) and pyridine (0.149 ml, 1.85 mmol) was added. The mixture was cooled to 273–278 K in ice-water bath. A separately prepared solution of chloroacetyl chloride (0.104 g, 0.925 mmol) in chloroform (5 ml) was added drop wise to the above mixture. The mixture was stirred for 3 h and solvent was evaporated to give a pink colored residue.Recrystallization from chloroform gave colorless needles with melting point of 471.15 K.

### S3. Refinement

The H-atoms were positioned geometrically (N—H = 0.86, C–H = 0.93–0.97 Å) and were included in the refinement in the riding model approximation, with *U*_iso_(H) = 1.2*U*_eq_(C, N).

### Crystal data

**Table:**

C_10_H_10_Cl_2_N_2_O_2_	*F*(000) = 536
*M**_r_* = 261.10	*D*_x_ = 1.588 Mg m^−^^3^
Monoclinic, *P*2_1_/*c*	Mo *K*α radiation, λ = 0.71073 Å
*a* = 4.5731 (4) Å	Cell parameters from 1685 reflections
*b* = 14.3260 (16) Å	θ = 1.9–26.0°
*c* = 16.7472 (15) Å	µ = 0.58 mm^−^^1^
β = 95.611 (5)°	*T* = 296 K
*V* = 1091.92 (18) Å^3^	Needle, colorless
*Z* = 4	0.40 × 0.22 × 0.16 mm

### Data collection

**Table:**

Bruker Kappa APEXII CCD diffractometer	2154 independent reflections
Radiation source: fine-focus sealed tube	1685 reflections with *I* > 2σ(*I*)
Graphite monochromator	*R*_int_ = 0.031
Detector resolution: 8.00 pixels mm^-1^	θ_max_ = 26.0°, θ_min_ = 1.9°
ω scans	*h* = −5→5
Absorption correction: multi-scan (*SADABS*; Bruker, 2005)	*k* = −17→14
*T*_min_ = 0.803, *T*_max_ = 0.911	*l* = −20→20
8308 measured reflections	

### Refinement

**Table:**

Refinement on *F*^2^	Primary atom site location: structure-invariant direct methods
Least-squares matrix: full	Secondary atom site location: difference Fourier map
*R*[*F*^2^ > 2σ(*F*^2^)] = 0.035	Hydrogen site location: inferred from neighbouring sites
*wR*(*F*^2^) = 0.083	H-atom parameters constrained
*S* = 1.03	*w* = 1/[σ^2^(*F*_o_^2^) + (0.0318*P*)^2^ + 0.392*P*] where *P* = (*F*_o_^2^ + 2*F*_c_^2^)/3
2154 reflections	(Δ/σ)_max_ < 0.001
145 parameters	Δρ_max_ = 0.25 e Å^−^^3^
0 restraints	Δρ_min_ = −0.28 e Å^−^^3^

### Special details

**Table:**

Geometry. All e.s.d.'s (except the e.s.d. in the dihedral angle between two l.s. planes) are estimated using the full covariance matrix. The cell e.s.d.'s are taken into account individually in the estimation of e.s.d.'s in distances, angles and torsion angles; correlations between e.s.d.'s in cell parameters are only used when they are defined by crystal symmetry. An approximate (isotropic) treatment of cell e.s.d.'s is used for estimating e.s.d.'s involving l.s. planes.
Refinement. Refinement of *F*^2^ against ALL reflections. The weighted *R*-factor *wR* and goodness of fit *S* are based on *F*^2^, conventional *R*-factors *R* are based on *F*, with *F* set to zero for negative *F*^2^. The threshold expression of *F*^2^ > σ(*F*^2^) is used only for calculating *R*-factors(gt) *etc*. and is not relevant to the choice of reflections for refinement. *R*-factors based on *F*^2^ are statistically about twice as large as those based on *F*, and *R*-factors based on ALL data will be even larger.

### Fractional atomic coordinates and isotropic or equivalent isotropic displacement parameters (Å^2^)

**Table:**

	*x*	*y*	*z*	*U*_iso_*/*U*_eq_	
Cl1	0.42275 (13)	0.31322 (5)	0.41502 (3)	0.0566 (2)	
Cl2	1.17253 (12)	−0.12690 (5)	0.49132 (3)	0.05386 (19)	
O1	0.5571 (3)	0.29789 (10)	0.23928 (9)	0.0451 (4)	
O2	1.2396 (3)	0.00838 (11)	0.36111 (8)	0.0451 (4)	
N1	0.5615 (3)	0.14134 (11)	0.26212 (9)	0.0310 (4)	
H1	0.4935	0.0990	0.2917	0.037*	
N2	0.8285 (3)	−0.03260 (11)	0.28396 (9)	0.0331 (4)	
H2	0.6706	−0.0656	0.2809	0.040*	
C1	0.2642 (4)	0.23927 (16)	0.33817 (13)	0.0420 (5)	
H1A	0.2243	0.1785	0.3602	0.050*	
H1B	0.0799	0.2655	0.3148	0.050*	
C2	0.4749 (4)	0.22930 (14)	0.27444 (11)	0.0318 (4)	
C3	0.7526 (4)	0.11071 (14)	0.20580 (10)	0.0297 (4)	
C4	0.8061 (4)	0.16327 (16)	0.13895 (12)	0.0392 (5)	
H4	0.7163	0.2211	0.1303	0.047*	
C5	0.9919 (5)	0.12981 (17)	0.08557 (12)	0.0441 (5)	
H5	1.0262	0.1653	0.0409	0.053*	
C6	1.1271 (4)	0.04451 (17)	0.09760 (12)	0.0428 (5)	
H6	1.2544	0.0228	0.0618	0.051*	
C7	1.0726 (4)	−0.00838 (15)	0.16302 (12)	0.0382 (5)	
H7	1.1621	−0.0664	0.1711	0.046*	
C8	0.8855 (4)	0.02408 (14)	0.21689 (11)	0.0301 (4)	
C9	1.0116 (4)	−0.03627 (13)	0.35130 (11)	0.0314 (4)	
C10	0.8997 (4)	−0.09830 (17)	0.41444 (13)	0.0451 (5)	
H10A	0.8221	−0.1553	0.3893	0.054*	
H10B	0.7399	−0.0669	0.4375	0.054*	

### Atomic displacement parameters (Å^2^)

**Table:**

	*U*^11^	*U*^22^	*U*^33^	*U*^12^	*U*^13^	*U*^23^
Cl1	0.0649 (4)	0.0620 (5)	0.0442 (3)	−0.0031 (3)	0.0113 (3)	−0.0083 (3)
Cl2	0.0561 (4)	0.0613 (4)	0.0443 (3)	0.0051 (3)	0.0057 (2)	0.0141 (3)
O1	0.0551 (9)	0.0273 (9)	0.0557 (9)	0.0023 (6)	0.0198 (7)	0.0054 (7)
O2	0.0390 (8)	0.0483 (10)	0.0478 (8)	−0.0158 (7)	0.0025 (6)	0.0089 (7)
N1	0.0331 (8)	0.0248 (10)	0.0368 (8)	−0.0025 (6)	0.0114 (7)	0.0037 (7)
N2	0.0272 (8)	0.0277 (9)	0.0448 (9)	−0.0047 (6)	0.0055 (7)	0.0056 (8)
C1	0.0354 (11)	0.0351 (13)	0.0574 (13)	−0.0016 (9)	0.0148 (9)	−0.0049 (11)
C2	0.0274 (9)	0.0287 (12)	0.0395 (10)	−0.0006 (8)	0.0040 (8)	0.0006 (9)
C3	0.0268 (9)	0.0299 (11)	0.0327 (9)	−0.0042 (8)	0.0052 (7)	−0.0024 (9)
C4	0.0439 (11)	0.0360 (13)	0.0389 (10)	0.0029 (9)	0.0106 (9)	0.0069 (10)
C5	0.0498 (13)	0.0494 (15)	0.0347 (10)	−0.0058 (11)	0.0122 (9)	0.0042 (10)
C6	0.0425 (12)	0.0494 (15)	0.0385 (11)	−0.0029 (10)	0.0139 (9)	−0.0100 (11)
C7	0.0351 (10)	0.0318 (12)	0.0484 (12)	0.0002 (9)	0.0071 (9)	−0.0068 (10)
C8	0.0269 (9)	0.0289 (11)	0.0345 (9)	−0.0055 (8)	0.0024 (8)	−0.0005 (9)
C9	0.0300 (10)	0.0237 (11)	0.0420 (10)	0.0011 (8)	0.0102 (8)	0.0015 (9)
C10	0.0410 (12)	0.0434 (14)	0.0507 (12)	−0.0061 (10)	0.0046 (10)	0.0123 (11)

### Geometric parameters (Å, º)

**Table:**

Cl1—C1	1.767 (2)	C3—C8	1.387 (3)
Cl2—C10	1.752 (2)	C3—C4	1.391 (3)
O1—C2	1.223 (2)	C4—C5	1.378 (3)
O2—C9	1.221 (2)	C4—H4	0.9300
N1—C2	1.343 (2)	C5—C6	1.375 (3)
N1—C3	1.417 (2)	C5—H5	0.9300
N1—H1	0.8600	C6—C7	1.375 (3)
N2—C9	1.338 (2)	C6—H6	0.9300
N2—C8	1.431 (2)	C7—C8	1.383 (3)
N2—H2	0.8600	C7—H7	0.9300
C1—C2	1.513 (3)	C9—C10	1.509 (3)
C1—H1A	0.9700	C10—H10A	0.9700
C1—H1B	0.9700	C10—H10B	0.9700
			
C2—N1—C3	127.11 (16)	C6—C5—C4	120.71 (19)
C2—N1—H1	116.4	C6—C5—H5	119.6
C3—N1—H1	116.4	C4—C5—H5	119.6
C9—N2—C8	122.45 (15)	C7—C6—C5	119.51 (19)
C9—N2—H2	118.8	C7—C6—H6	120.2
C8—N2—H2	118.8	C5—C6—H6	120.2
C2—C1—Cl1	109.02 (14)	C6—C7—C8	120.4 (2)
C2—C1—H1A	109.9	C6—C7—H7	119.8
Cl1—C1—H1A	109.9	C8—C7—H7	119.8
C2—C1—H1B	109.9	C7—C8—C3	120.32 (18)
Cl1—C1—H1B	109.9	C7—C8—N2	119.55 (18)
H1A—C1—H1B	108.3	C3—C8—N2	120.13 (16)
O1—C2—N1	124.80 (17)	O2—C9—N2	123.29 (18)
O1—C2—C1	120.69 (19)	O2—C9—C10	123.94 (17)
N1—C2—C1	114.50 (17)	N2—C9—C10	112.73 (16)
C8—C3—C4	118.89 (17)	C9—C10—Cl2	112.76 (14)
C8—C3—N1	118.63 (16)	C9—C10—H10A	109.0
C4—C3—N1	122.47 (18)	Cl2—C10—H10A	109.0
C5—C4—C3	120.1 (2)	C9—C10—H10B	109.0
C5—C4—H4	119.9	Cl2—C10—H10B	109.0
C3—C4—H4	119.9	H10A—C10—H10B	107.8
			
C3—N1—C2—O1	−2.1 (3)	C6—C7—C8—N2	179.12 (17)
C3—N1—C2—C1	178.87 (16)	C4—C3—C8—C7	1.2 (3)
Cl1—C1—C2—O1	−58.8 (2)	N1—C3—C8—C7	179.76 (16)
Cl1—C1—C2—N1	120.23 (16)	C4—C3—C8—N2	−178.35 (17)
C2—N1—C3—C8	161.13 (17)	N1—C3—C8—N2	0.2 (2)
C2—N1—C3—C4	−20.4 (3)	C9—N2—C8—C7	81.8 (2)
C8—C3—C4—C5	−0.9 (3)	C9—N2—C8—C3	−98.6 (2)
N1—C3—C4—C5	−179.38 (18)	C8—N2—C9—O2	0.4 (3)
C3—C4—C5—C6	−0.2 (3)	C8—N2—C9—C10	178.10 (17)
C4—C5—C6—C7	0.9 (3)	O2—C9—C10—Cl2	−17.4 (3)
C5—C6—C7—C8	−0.6 (3)	N2—C9—C10—Cl2	164.89 (15)
C6—C7—C8—C3	−0.5 (3)		

### Hydrogen-bond geometry (Å, º)

**Table:**

*D*—H···*A*	*D*—H	H···*A*	*D*···*A*	*D*—H···*A*
N1—H1···O2^i^	0.86	2.16	3.003 (2)	168
N2—H2···O1^ii^	0.86	2.23	3.004 (2)	150
C1—H1*A*···O2^i^	0.97	2.44	3.333 (3)	153

Symmetry codes: (i) *x*−1, *y*, *z*; (ii) −*x*+1, *y*−1/2, −*z*+1/2.
