# Supplementary figures and images for: Crystal structure of N,N′-(1,2-phenyl­ene)bis­(2-chloro­acetamide)
Source: Acta Crystallogr E Crystallogr Commun. 2015 Jan 14;71(Pt 2):o108. doi: 10.1107/S2056989015000304 (PMC4384540; doi:10.1107/S2056989015000304)

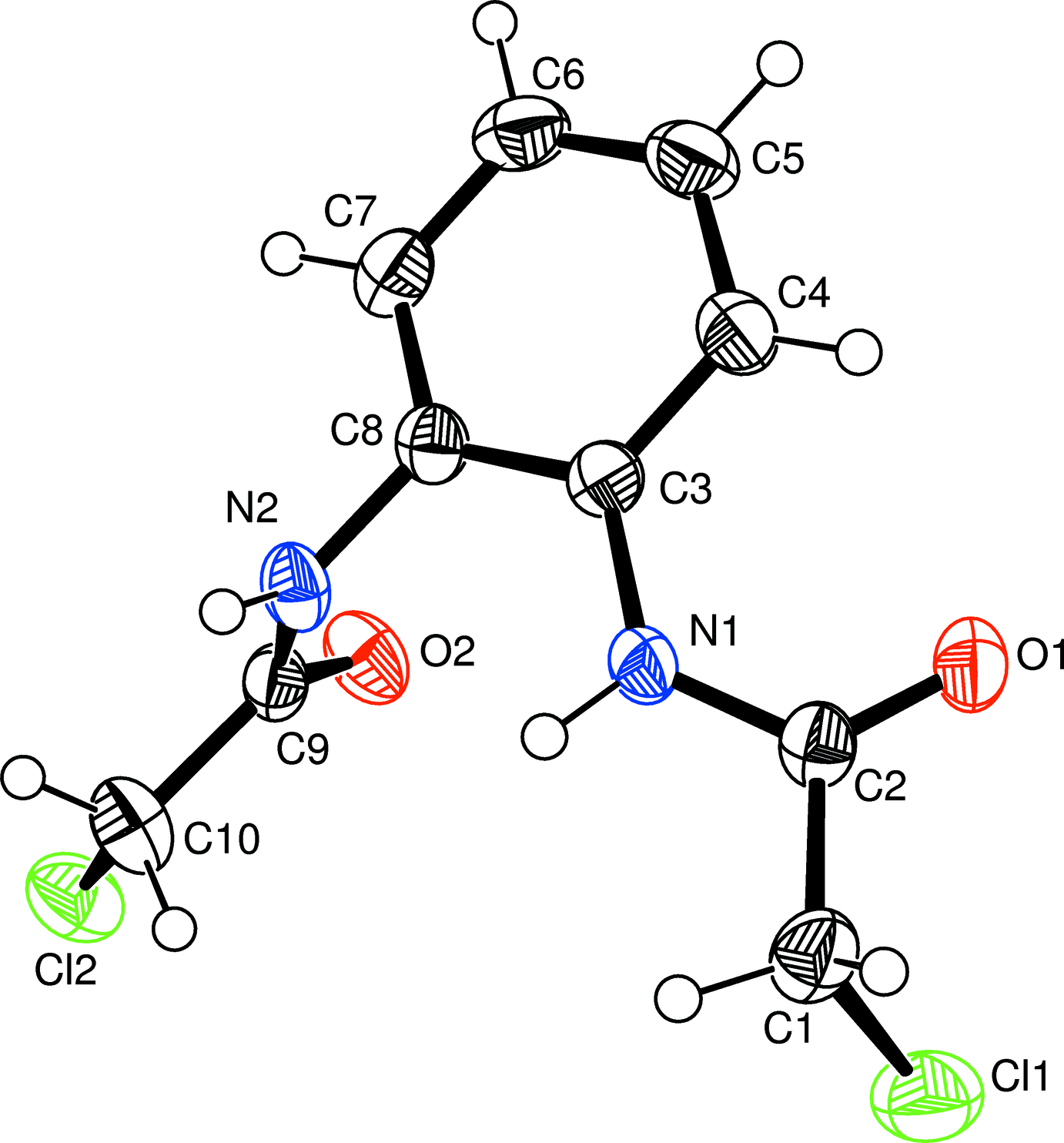

Supplement: Supplementary file 4 [file e-71-0o108-fig1.tif]

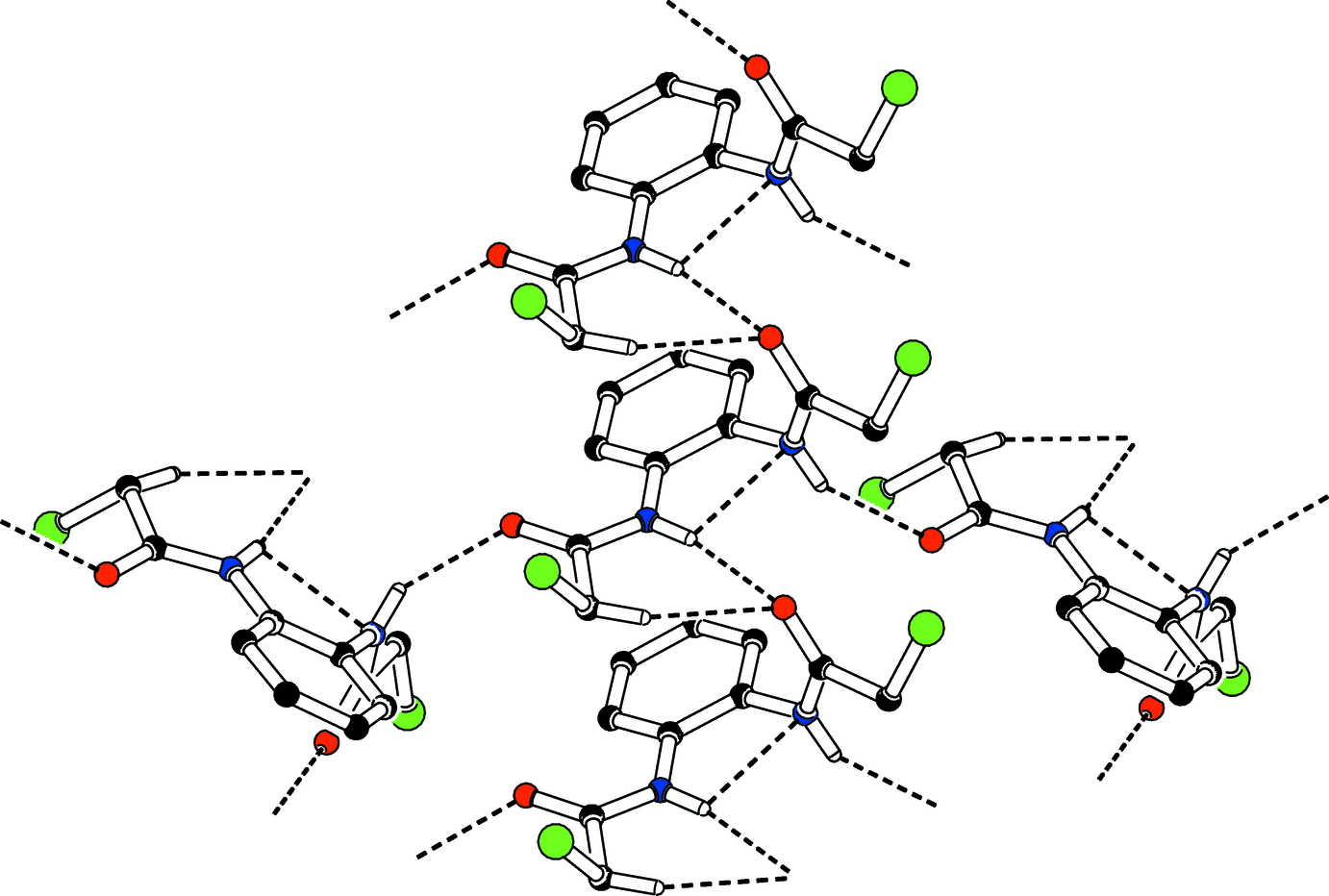

Supplement: Supplementary file 5 [file e-71-0o108-fig2.tif]
